# Autoimmune-Associated Livedoid Vasculopathy: Response to Immunomodulatory Therapy

**DOI:** 10.7759/cureus.92748

**Published:** 2025-09-19

**Authors:** Thanda Aung, Jenice X Cheah

**Affiliations:** 1 Rheumatology, University of California Los Angeles David Geffen School of Medicine, Los Angeles, USA

**Keywords:** hydroxychloroquine, intravenous immunoglobulins (ivig), livedoid vasculopathy, positive antinuclear antibody, undifferentiated connective tissue disease

## Abstract

Livedoid vasculopathy (LV) is a rare, chronic occlusive dermopathy causing recurrent painful ulcerations, impaired healing, and significant morbidity. Historically considered a thrombotic microangiopathy, emerging evidence suggests an autoimmune or mixed pathogenesis in some patients. We present the case of a 55-year-old woman with LV and serologic features of undifferentiated connective tissue disease (UCTD) who achieved complete, durable remission using intravenous immunoglobulin (IVIG) and hydroxychloroquine (HCQ). After 16 years of refractory disease, including failure of multiple anticoagulant, antiplatelet, and immunosuppressive regimens, her ulcers resolved completely and have not recurred for more than two years. This case underscores the need to evaluate for autoimmune features in LV, highlights IVIG as an effective treatment, and demonstrates that HCQ may serve as a valuable adjunct in refractory autoimmune-associated LV.

## Introduction

Livedoid vasculopathy (LV) is an uncommon thrombo-occlusive microangiopathy of the dermal vasculature, with an estimated prevalence of approximately one in 100,000 [1]. It typically affects middle-aged women and presents with recurrent painful purpuric lesions progressing to deep ulcerations that heal with stellate porcelain-white scars (atrophie blanche) [1,2]. Histopathologic features show fibrin deposition and vascular thrombosis with minimal inflammatory infiltrate [2]. Traditionally regarded as a coagulation disorder rather than an inflammatory vasculitis, LV has recently been linked to autoimmune mechanisms, with approximately 30% of patients testing antinuclear antibody (ANA)-positive and many cases occurring in association with systemic autoimmune rheumatic diseases (SARD) [3-5]. Management remains challenging. Conventional therapy emphasizes anticoagulation, but complete remission is uncommon [2,6]. Direct oral anticoagulants (DOACs) show promise in recent series [7,8]. Immunomodulatory agents, particularly intravenous immunoglobulin (IVIG), have emerged as effective in refractory LV, achieving high remission rates in multiple reports [9-11]. The addition of disease-modifying antirheumatic drugs such as hydroxychloroquine (HCQ) may further benefit patients with coexisting autoimmune features [12].

## Case presentation

A 55-year-old woman presented with a 16-year history of recurrent painful ulcerations on both lower extremities. The lesions evolved from purpuric macules to deep ulcers and eventually healed with stellate porcelain-white scars. Pain was severe (8-9/10), described as burning and stabbing, particularly at night and with ambulation. Her history included heterozygous prothrombin G20210A mutation, hypothyroidism controlled with levothyroxine, and mild hypertension. Family history revealed systemic lupus erythematosus in her sister and rheumatoid arthritis in a maternal aunt. A prior skin biopsy confirmed LV without inflammatory vasculitis, but pathology slides were unavailable because the biopsy was performed at an outside facility.

**Figure 1:**
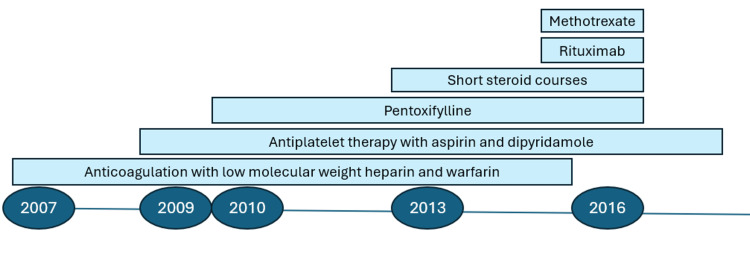
The patient's treatment history prior to initial evaluation at our center in 2018.

Over more than a decade, the patient underwent multiple therapies with minimal benefit (Figure 1). Anticoagulation with low molecular weight heparin followed by warfarin (2007-2015) did not prevent frequent flares. Antiplatelet therapy with aspirin and dipyridamole (2009-2018), pentoxifylline (2010-2016), and topical wound treatments offered little improvement. Short corticosteroid courses (2013-2017) provided temporary relief. Rituximab (2016) was ineffective, and methotrexate (2016) was discontinued due to hepatotoxicity without clinical benefit.

At initial evaluation in our clinic (2018), she had multiple stages of active LV lesions from mid-calf to the feet, including painful ulcerations, purpuric macules, and extensive atrophie blanche. Laboratory findings included ANA 1:1280 (speckled), anti-Sjögren's syndrome A (Ro) antibodies (anti-Ro/SSA) positivity, erythrocyte sedimentation rate (ESR)28 mm/hr, and confirmed heterozygosity for prothrombin G20210A mutation (Table 1). Other thrombophilia markers, including protein C/S deficiency and antiphospholipid antibodies, were negative. In conjunction with polyarthralgia and photosensitivity, these findings supported a diagnosis of undifferentiated connective tissue disease (UCTD) overlapping with LV. 

**Table 1:** Summary of the patient's laboratory results from 2018-2022 ANA: anti-nuclear antibody; SSA: anti-Sjögren's syndrome A (Ro) antibodies; ACE: angiotensin-converting enzyme; IFA: immunofluorescence assay; ESR: erythrocyte sedimentation rate; CRP: c-reactive protein; c-ANCA: cytoplasmic anti-neutrophil cytoplasmic antibodies; p-ANCA: perinuclear anti-neutrophil cytoplasmic antibodies; C3: complement component 3; C4: complement component 4

Test	Result	Reference Range
ANA	1:1280 (speckled pattern)	<1:40 negative
Anti-Ro/SSA	1.4	<1.0 negative
ESR	36-63 mm/hr	0-20 mm/hr
CRP	Within normal limits	<3.0 mg/L
C3	112-116 mg/dL	90-180 mg/dL
C4	20-26 mg/dL	10-40 mg/dL
Anti-dsDNA (ELISA)	≤200	<200 negative
Anti-dsDNA (IFA)	<1:10	<1:10 negative
c-ANCA	<1:20	<1:20 negative
p-ANCA	<1:20	<1:20 negative
Cryoglobulins	Not detected	Negative
Serum ACE	Normal	8-53 U/L
Prothrombin gene mutation	Heterozygous G20210A	Negative
Factor V Leiden	Negative	Negative
Protein C	Normal	70-140%
Protein S	Normal	65-140%

Given the refractory disease course, IVIG was initiated at 2 g/kg every six weeks. Ulcer healing improved markedly, though intermittent painful flares continued. In 2022, HCQ (200 mg twice daily) was added to address autoimmune features. The combination led to complete cessation of disease flares for over 24 months, resolution of all active ulcerations without recurrence, successful reduction of IVIG dosing interval to every eight weeks, and significant quality-of-life improvement without treatment-related adverse effects.

**Figure 2:**
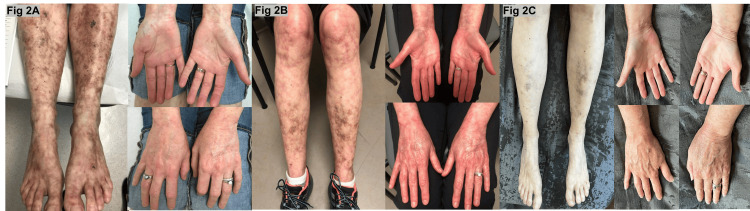
Serial clinical images of livedoid vasculopathy showing progressive improvement. (A) Rash in 2018 prior to initiation of IVIG; (B) Rash in 2021 after approximately three years of IVIG; (C) Rash in 2025 after three years of HCQ and seven years of IVIG. IVIG: intravenous immunoglobulin; HCQ: hydroxychloroquine

Serial clinical photographs demonstrate progressive improvement over time (Figure 2). Figure 2A (2018) shows active purpuric macules and ulcerations prior to initiation of IVIG. Figure 2B (2021) shows marked healing after approximately three years of IVIG therapy. Figure 2C (2025) demonstrates near-complete resolution of lesions with mature stellate scars after three years of HCQ and seven years of IVIG.

## Discussion

The pathogenesis of LV is heterogeneous, involving both thrombotic and inflammatory mechanisms. Approximately 50% of patients have identifiable thrombophilic disorders such as factor V Leiden, prothrombin gene mutations, protein C/S deficiency, or antiphospholipid antibodies [2,3,5]. However, 20% to 30% have no identifiable coagulation abnormality, implicating immune-mediated vascular injury [2,3,5]. Autoimmune features, including ANA positivity and overlap with systemic lupus erythematosus, Sjögren's syndrome, or rheumatoid arthritis, support the concept of an "autoimmune-associated LV" subtype [3,4].

Traditional anticoagulation achieves partial control in many patients but rarely induces complete remission [2,6]. Recent reports suggest DOACs such as rivaroxaban may be effective in some cases [7,8]. Immunomodulatory therapy has proven valuable in refractory LV. IVIG exerts pleiotropic effects, including antibody neutralization, complement inhibition, modulation of Fc receptor-mediated inflammation, and prevention of thrombosis [9,10]. Case series demonstrate complete or near-complete remission in up to 70% of refractory LV patients [9-11].

Our patient’s findings suggest an autoimmune-associated subtype of LV. IVIG can induce remission in refractory LV, although HCQ’s role remains unclear [8,10]. However, HCQ’s immunomodulatory effects may mitigate the underlying inflammation in this LV subtype, as observed in our case. 

This case highlights the importance of evaluating for SARD in LV patients and considering combination anti-thrombotic and immunomodulatory treatment; adding HCQ to IVIG may enhance long-term disease control in autoimmune-associated LV [12]. Our patient's sustained remission for over two years on IVIG plus HCQ supports a treatment paradigm that addresses both thrombotic and autoimmune mechanisms simultaneously.

## Conclusions

Clinicians should evaluate refractory LV patients for underlying autoimmune features. In selected cases, combination immunomodulatory therapy can achieve durable remission where standard anticoagulation alone is insufficient. The dramatic response to IVIG plus HCQ in this patient supports an evolving concept of autoimmune-associated LV and underscores the value of individualized therapy tailored to disease mechanisms.
